# A novel *in situ* stress measurement method based on acoustic emission Kaiser effect: a theoretical and experimental study

**DOI:** 10.1098/rsos.181263

**Published:** 2018-10-24

**Authors:** Xin Bai, Dong-ming Zhang, Hao Wang, Shu-jian Li, Zi Rao

**Affiliations:** 1 State Key Laboratory of Coal Mine Disaster Dynamic and Control, Chongqing University, Chongqing 400044, People's Republic of China; 2 College of Resources and Environmental Science, Chongqing University, Chongqing 400044, People's Republic of China; 3 College of Mathematics and Statistics, Chongqing University, Chongqing 400044, People's Republic of China; 4 Shamushu Coal Mine, Sichuan Furong Group Industry Co., Ltd, Gongxian 644500, People's Republic of China

**Keywords:** *in situ* stress, Kaiser effect, acoustic emission, uniaxial compression testing, mechanical properties

## Abstract

Measurement of *in situ* stress is critical to understand the deformation and destruction of the underground space surrounding rock, and dynamic disaster of the coal mine. At present, with the increasing depth of mining, *in situ* stress parameters are more and more important for coal mine. In this paper, a novel method for *in situ* stress measurement with Kaiser effect was studied and applied in the Baijiao coal mine. First, we presented a comprehensive analysis method for the identification of Kaiser effect point. Then, a calculation method for *in situ* stress measurement based on the Kaiser effect on acoustic emissions was suggested. After that, the *in situ* stress test of Baijiao coal mine is taken as the research object, an experiment using acoustic emissions monitoring during uniaxial compression testing was performed to investigate the mechanical properties and acoustic emissions characteristics. Finally, *in situ* stress of the study area was calculated using the novel calculation method above and calculation results were verified using stress relief method and hydraulic fracturing method. The results showed that the calculation results obtained using the proposed method were valid and credible. Therefore, the calculation method for *in situ* stress measurement and the proposed comprehensive analysis method using the Kaiser point could be applied for *in situ* stress testing using the Kaiser effect method.

## 1 Introduction

*In situ* stress is the natural stress field existing in strata prior to any man-made disturbance and is critical to the study of coal mine underground space surrounding rock support [1], coal and gas outbursts [2], coalbed methane extraction [3,4] and rock burst [5]. In recent years, with the increasing depth of mining, resulting in high *in situ* stress, engineering geological environment brings great challenges to deep mine roadway surrounding rock support and deep mine dynamic disaster prevention [6]. Therefore, it is of great significance for the deep mine construction and its safety production to measurement of the *in situ* stress of the deep mine [7]. Over the past 40 years, a variety of techniques and method for *in situ* stress measurement has been extensively studied and used, such as hydraulic fracturing (HF) [8,9], the stress relief method [10], acoustic emission (AE) [11,12] and borehole breakout [13]. Among these stress measurement techniques, the methods of stress relief and HF have been widely applied. However, the stress relief method and HF method were considered to be unexpectedly low for absolute value of principal stress [13]. And stress relief method requires high precision for drilling construction, so is only applicable to the open space where the tester can enter and can construct the measurement holes [14,15]. It is not suitable for formation original stress measurement with depth exceeding 1000 m [16]. Hydraulic fracturing and borehole breakout were researched as a potential method for determining *in situ* stress at depth in laboratory and field tests [17,18]. Usually, an orientation of one of the principal stresses is assumed to be vertical in stress measurement. Accordingly, the equation can be two-dimensional. And information of three-dimensional stress distributions is very important for reliable underground structural designing, even though the information is only for narrow limited area [15,19–21]. Thus, a scientific and effective *in situ* measurement method using laboratory measurements is needed.

The Kaiser effect is a potentially promising method for determining the stress state at depth and in remote regions [19,22,23]. Under cyclic loading and unloading some materials produce little or no AE until the stress of the previous cycle is exceeded. This is referred to as the Kaiser ‘stress memory’ effect [24]. The best understood and established manifestation of the Kaiser effect occurs under uniaxial compression where the Kaiser effect can be used to measure the previous maximum loading of the material [14]. The Kaiser effect has been shown to occur in many rock types [25] and has been suggested as a way to measure *in situ* stresses, thus with possible application to many underground structural designs [12,19,23,26–29]. The method of Kaiser effect *in situ* stress measurement mainly includes the following steps: field drilling sampling, rock specimen processing, uniaxial compression and AE experiments, Kaiser effect identification, principal stress calculation. According to the previous studies, this method has three problems that have not been solved completely as of today [22,30]. First, the existing Kaiser effect identification method is based on the trend of the AE count [27,31,32], but the method has some subjectivity in the application process [33]. So a scientific and effective method for determining the Kaiser effect point needs to be established. Second, the existing principal stress calculation method of Kaiser effect method requires the rock specimen to be collected in the special direction [32,34], and because the engineering construction site conditions are complicated, it will inevitably cause the sampling angle deviation, which may cause the calculation result deviation [35]. Therefore, it is necessary to establish a new principal stress calculation method that has no special requirements for the sampling drilling angle. Third, most of the existing research assumes that the rock specimens are homogeneous [26,29,34], during the process of Kaiser effect identification. And the internal structure of the rock specimens has never been considered by the researchers during the Kaiser effect point identification process. However, the research of Zhang *et al.* [36] and Sun *et al.* [37] shows that the layered structure within the rock specimens has great influence on the AE characteristics and Kaiser effect. And resources such as coal, oil and natural gas are mainly stored in layered sedimentary rocks [38]. Therefore, it is not negligible for the AE characteristics and Kaiser effect identification of the influence by layered structure within the rock specimen.

For the above three problems, in this paper, considering the influence of the internal structure of rocks on the discrimination of Kaiser effect points, a method was established for comprehensive determination of Kaiser effect point. Then, a principal stress calculation method was established based on the principle of elasticity. After that, AE monitoring of rock specimens under uniaxial compression conditions was performed in laboratory experiments, and the failure, AE characteristics of the rock specimens were analysed. Finally, the test results were analysed and verified by stress relief method and hydraulic fracturing method, and the main factors controlling the *in situ* stress in the measurement site were analysed based on its geological structure.

## 2. Principles of the novel *in situ* stress measurement method

### 2.1. Comprehensive method for Kaiser effect identification

**Figure 1.**
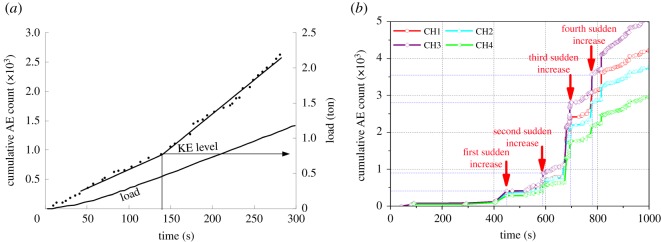
(*a*) Estimation of Kaiser Effect (KE) from ‘total AE count-time’ graph [31]; (*b*) suspicious Kaiser effect in ‘total AE count-time’ graph of rock specimen.

The Kaiser effect identification is a key step for *in situ* stress measurement with Kaiser effect method. In the Kaiser effect identification, Hayashi *et al.* [31] suggested that Kaiser effect levels obtained from the ‘cumulative AE count stress’ response could be used to obtain *in situ* stress (figure 1*a*). Essentially, this method is based on the ‘take-off point’ of AE count. After that, Qin *et al.* [27], Lavrov [22] and Jiang *et al.* [32] used this method to carry on the Kaiser effect judgement. Although this method has the characteristics that the data processing process is simple, there is no specific standard in the application process. Due to the sudden increase of AE counts in different degrees, there may be a number of suspected Kaiser effect points, as shown in figure 1*b*, so using the ‘take-off point’ to identify the Kaiser effect has great subjectivity, easily affected by human factors [33].

In recent years, scholars have performed a series of in-depth studies and proposed a series of methods for the Kaiser effect point determination. The tangent-intersection method was suggested by Boyce *et al.* [39], based on the curve of AE parameters with time. Hardy [40] and Hughson & Crawford [41] suggested determining the Kaiser effect point using mathematical statistics, felicity ratio and maximum curvature methods, respectively. Zhao *et al.* [42] proposed a Kaiser effect point determination method based on the G-P algorithm for calculating the AE energy correlation fractal dimension. These results contributed to the quantitative determination of the Kaiser effect, but the analysis methods are more complex, leading to difficulties with using these processes. Therefore, it is necessary to devise a new method for Kaiser effect identification in the AE method of *in situ* stress measurement.

**Figure 2.**
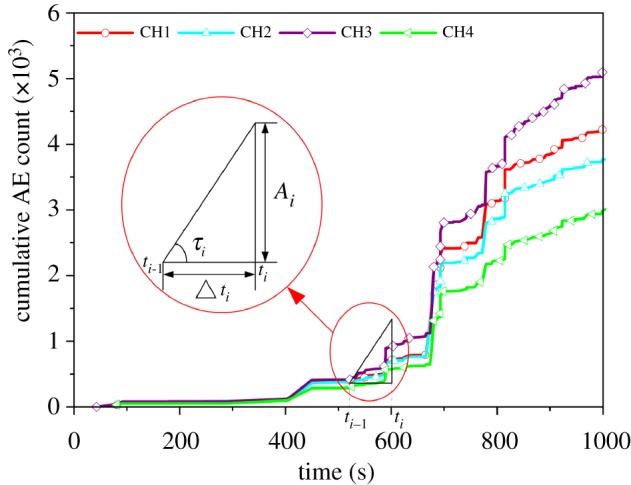
Schematic diagram of related parameters in equation (2.1).

The present study suggested combining the concepts of the Kaiser effect to derive a comprehensive method for Kaiser effect identification. The Kaiser effect on AE is caused by the reloading of brittle materials after stress release. That is, when the axial load is less than the maximum historical principal stress, only a small number of AE signals are generated, and when the axial load exceeds the historical maximum principal stress, many AE signals begin to be generated [24]. Therefore, the AE signals generated by rock specimens near the Kaiser effect have obvious time and quantity characteristics that manifest as increases in the slope and inclination of the curve representing the cumulative AE count over time. And the slope and inclination of the curve can be processed and analysed using equation (2.1):

2.1 $$\left.\begin{array}{rl} \Delta t_{i} & =t_{i}-t_{i-1},\\ k & =\frac{A_{i}}{\Delta t_{i}}\\ \;\mathrm{and}\;\tau_{i} & =\arctan k_{i} \end{array}\right\}$$

wherein, *t_i_* are the time of AE counts; Δ*t_i_* is the time difference between these two adjacent AE counts, *A_i_* is the AE count; *k_i_* and *τ_i_* are the slope and the slope angle, respectively, of the curve at time *i*. As shown in figure 2, Δ*t_i_* is a parameter reflecting the time characteristics of the AE counts during the compression of the rock specimen. The smaller the Δ*t_i_* value, the more frequent the AE count is during the corresponding time interval. And if *τ_i_* is close to 90°, the AE count for the corresponding time increased dramatically.

When comparing the changes of the two parameters, Δ*t_i_* and *τ_i_*, if the value of Δ*t_i_* is smaller and the value of *τ_i_* is close to 90° at the same time, this indicates that the cumulative AE count at this time is greatly increased, and the AE count frequency is high, which is in accordance with the definition of the Kaiser effect. Therefore, the Kaiser effect point can be identified through comparison of Δ*t_i_* and *τ_i_*. The detailed procedures of Kaiser effect identification are as follows: (i) According to the stress—cumulative AE count-time curve, the time interval of suspected Kaiser effect can be preliminarily determined. (ii) The parameters of Δ*t_i_* and *τ_i_* are obtained by analysing the cumulative acoustic emission count over time using equation (2.1). (iii) The relationship curves of stress, Δ*t_i_*, *τ_i_* and time are obtained; according to the value of Δ*t_i_* and *τ_i_* the time of Kaiser effect can be determined. The corresponding stress value for this time is the historical maximum stress of the rock specimen in that direction.

### 2.2. The principle of principal stress calculation of *in situ* stress

The rock is assumed to be subjected to a three-dimensional stress field with components *σ_x_*, *σ_y_*, *σ_z_*, *τ_xy_*, *τ_xz_* and *τ_yz_* acting at infinity and defined in an *x*, *y*, *z* coordinate system attached to the measurement site. As shown by Amadei [16] and Zoback [18] if the six stress components (*σ_x_*, *σ_y_*, *σ_z_*, *τ_xy_*, *τ_xz_*, *τ_yz_*) of *in situ* stress in the *x*, *y*, *z* coordinate system can be obtained, the principal stress (including size and direction) of *in situ* stress can be calculated indirectly. And Sjöberg *et al.* [10] put forward that measurements from at least six independent directions are required to determine the stress tensor (which has six components), in the process of principal stress calculation method study.

Therefore, the Kaiser effect method can be used to obtain the historical maximum stress of the rock specimen in the relevant direction [14]. And the principal stress of *in situ* stress can be calculated based on the elastic mechanics theory [32]. For example, Chen *et al.* [34] proposed a calculation method of *in situ* stress by Kaiser effect method. The results showed the six stress components (*σ_x_*, *σ_y_*, *σ_z_*, *τ_xy_*, *τ_xz_*, *τ_yz_*) of the principal stress can be obtained by the Kaiser effect of six independent directions (*x*, *y*, *z*, *xy*45°, *xz*45°, *yz*45°) rock specimens, and then the size and direction of *in situ* stress can be calculated. However, as in the preceding analysis, we found that the existing principal stress calculation method for *in situ* stress measurement using the AE Kaiser effect method required the rock specimens to be sampled from six specific directions. During rock specimen collection, these sampling conditions are often not completely satisfied because site conditions can be complicated, especially the *xy*45°, *xz*45°, *yz*45°. Zhang & Chen's [35] research showed that the extraction angle deviation affects the *in situ* stress measurement results from the AE Kaiser effect method.

In this paper, based on the theory of elastic mechanics and previous research [15,32] and considering the relationships between the plane stresses, an improved calculation method for *in situ* stress measurements using the AE Kaiser effect is suggested. The method can satisfy the stress tensor calculation when the sampling angle deviates, thus avoiding the deviation in the principal stress calculation results caused by the deviation of the extracting angles of the rock specimens.

#### 2.2.1. Shear stress analysis of a tetrahedral element

**Figure 3.**
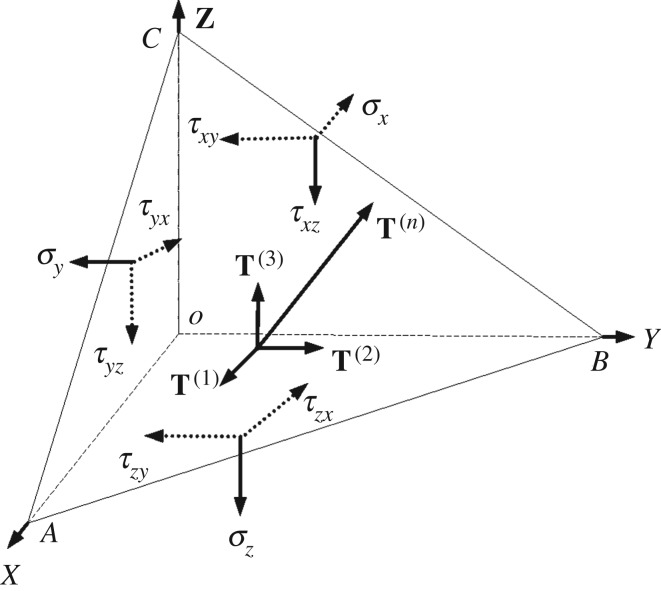
Stress distribution of tetrahedron [44].

According to the basic principles of elastic mechanics [43], the stress distribution on the tetrahedron *OABC* is shown in figure 3. The exterior normal of surface *ABC* is **n**, and the directional cosines of **n** are *α_x_*, *α_x_* and *α_z_*. The area of surface *ABC* is *S*, and the stress vector is **T**^(*n*)^. Thus, *Sα_x_*, *Sα_y_* and *Sα_z_* are the areas of faces *OBC*, *OAC* and *OAB*, respectively, and −**T**^(1)^, −**T**^(2)^ and −**T**^(3)^ are the stress vectors of faces *OBC*, *OAC* and *OAB*, respectively.

The force equilibrium equation for the tetrahedral element *OABC* is [44]:

2.2 $$T^{\left(n\right)}S-(T^{\left(1\right)}S_{1}+T^{\left(2\right)}S_{2}+T^{\left(3\right)}S_{3})+f\cdot Sh/3=0$$

where **f** is the volume force of the tetrahedral element *OABC*; *h* is the vertical distance from point *O* to surface *ABC*; and *Sh*/3 is the volume of the tetrahedral element *OABC*. When the tetrahedron is infinitely reduced, the last term of the above equation is an infinitesimal quantity, which is neglected, and the stress vector **T**^(*n*)^ satisfies the following equation:

2.3 $$T^{\left(n\right)}=T^{\left(1\right)}\alpha_{x}+T^{\left(2\right)}\alpha_{y}+T^{\left(3\right)}\alpha_{z}$$

The stress vectors **T**^(1)^, **T**^(2)^ and **T**^(3)^ of face *ABC* can be decomposed into a stress component matrix along the axes of the *X*, *Y* and *Z* directions as follows:

2.4 $$\left.\begin{array}{c} T^{\left(1\right)}=\sigma_{x}e_{x}+\tau_{yx}e_{y}+\tau_{zx}e_{z}\\ T^{\left(2\right)}=\tau_{xy}e_{x}+\sigma_{y}e_{y}+\tau_{zy}e_{z}\\ T^{\left(3\right)}=\tau_{xz}e_{x}+\tau_{yz}e_{y}+\sigma_{z}e_{z} \end{array}\right\}$$

Using equation (2.4), **T**^(*n*)^ can be expressed as follows:

2.5 $$T^{\left(n\right)}=T^{\left(1\right)}e_{x}+T^{\left(2\right)}e_{y}+T^{\left(3\right)}e_{z}$$

where,

2.6 $$\left.\begin{array}{c} T^{\left(1\right)}=\sigma_{x}\alpha_{x}+\tau_{yx}\alpha_{y}+\tau_{zx}\alpha_{z}\\ T^{\left(2\right)}=\tau_{xy}\alpha_{x}+\sigma_{y}\alpha_{y}+\tau_{zy}\alpha_{z}\\ T^{\left(3\right)}=\tau_{xz}\alpha_{x}+\tau_{yz}\alpha_{y}+\sigma_{z}\alpha_{z} \end{array}\right\}$$

Combining equations (2.5) and (2.6), gives [32]:

2.7 $$T^{\left(n\right)}=\sigma_{x}\alpha_{x}^{2}+\sigma_{y}\alpha_{y}^{2}+\sigma_{z}\alpha_{z}^{2}+\tau_{xy}\alpha_{x}\alpha_{y}+\tau_{xz}\alpha_{x}\alpha_{z}+\tau_{yx}\alpha_{y}\alpha_{x}+\tau_{yz}\alpha_{y}\alpha_{z}+\tau_{zx}\alpha_{z}\alpha_{x}+\tau_{zy}\alpha_{z}\alpha_{y}$$

From equation (2.7), the stress matrix $\left(\begin{array}{ccc} \sigma_{x} & \tau_{yx} & \tau_{zx}\\ \tau_{xy} & \sigma_{y} & \tau_{zy}\\ \tau_{xz} & \tau_{yz} & \sigma_{z} \end{array}\right)$ of the tetrahedral element *OABC* can be solved for the stress vector on face *ABC*.

**Figure 4.**
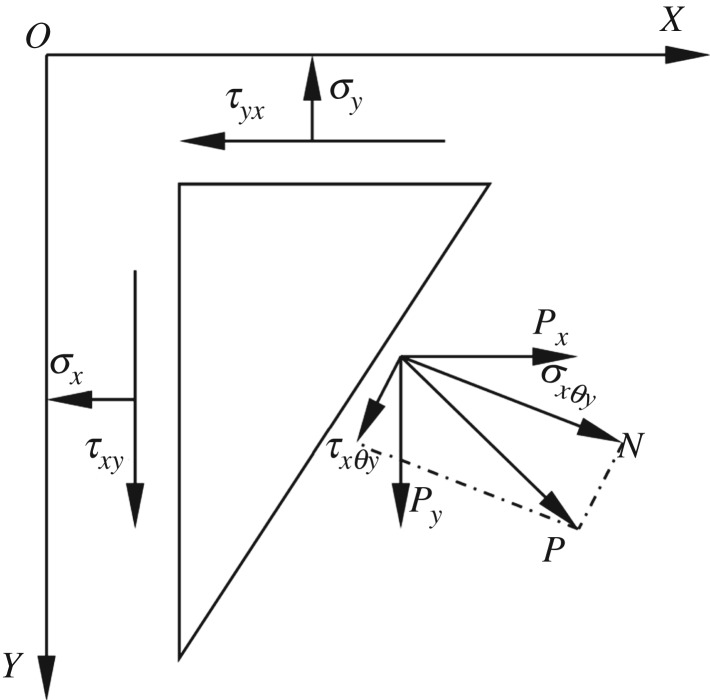
Sketch of stress state at the face *OXY*.

Assuming that the rock is a continuous and homogeneous elastomer [43], that is $\tau_{xy}=\tau_{yx},\;\tau_{xz}=\tau_{zx},\;\tau_{yz}=\tau_{zy}$, these six stress components can be obtained to calculate the stress vector **T**^(*n*)^ on face *ABC*. For obtaining the shear stresses $\tau_{xy,}\tau_{xz}\;\mathrm{and}\;\tau_{yz}$ of faces *OXY*, *OXZ* and *OYZ*, respectively, the stress distribution on face *OXY* is shown in figure 4.

As shown in figure 4, the following relationship exists between the principal stress, *σ_xθ_*_y_, and the shear stress, *τ_xy_*, in the cross section of face *OXY* at an angle of *θ* from the *X* axis:

2.8 $$\sigma_{x\theta y}=\sigma_{x}\cos^{2}\theta +\sigma_{y}\sin^{2}\theta +2\tau_{xy}\cos\theta \sin\theta$$

Therefore, the following relations can be obtained:

2.9 $$\left.\begin{array}{c} \tau_{xy}=\frac{\sigma_{x\theta y}-\sigma_{x}\;\cos^{2}\theta -\sigma_{y}\;\sin^{2}\theta }{2\;\cos\theta \;\sin\theta }\\ \tau_{xz}=\frac{\sigma_{x\gamma z}-\sigma_{x}\;\cos^{2}\gamma -\sigma_{z}\;\sin^{2}\gamma }{2\;\cos\gamma \;\sin\gamma }\\ \tau_{yz}=\frac{\sigma_{y\psi z}-\sigma_{y}\;\cos^{2}\psi -\sigma_{z}\;\sin^{2}\psi }{2\;\cos\psi \;\sin\psi } \end{array}\right\}$$

Therefore, the shear stress can be obtained for any cross section of the space when *σ_xθy_*, *σ_xγz_*, *σ_yψz_*, *θ*, *γ* and *ψ* are known.

#### 2.2.2. Principal stress calculation

If the stress vector **T^(n)^** on face *ABC* of tetrahedral element *OABC* is consistent with the exterior normal **n**, the shear stress on this face is zero. The stress on this face is called the principal stress **σ**, its directional cosines are *α_x_*, *α_y_* and *α_z_*_,_ and, at some point in the space, there must be three principal vertical stresses, represented by **σ_1_**, **σ_2_** and **σ_3_** (generally, *σ*_1_ ≥ *σ*_2_ ≥ *σ*_3_), which are referred to as the maximum principal stress (**σ_1_**), intermediate principal stress (**σ_2_**) and the minimum principal stress (**σ_3_**) [45].

The principal stress *σ* on face *ABC* satisfies the following equation (2.10):

2.10 $$\left.\begin{array}{c} T^{\left(1\right)}=\sigma \alpha_{1}\\ T^{\left(2\right)}=\sigma \alpha_{2}\\ T^{\left(3\right)}=\sigma \alpha_{3} \end{array}\right\}$$

Combining equations (2.10) and (2.6), we have

2.11 $$\left.\begin{array}{c} (\sigma_{x}-\sigma )\alpha_{1}+\tau_{yx}\alpha_{2}+\tau_{zx}\alpha_{3}=0\\ \tau_{xy}\alpha_{1}+(\sigma_{y}-\sigma )\alpha_{2}+\tau_{zy}\alpha_{3}=0\\ \tau_{xz}\alpha_{1}+\tau_{yz}\alpha_{2}+(\sigma_{z}-\sigma )\alpha_{3}=0 \end{array}\right\}$$

Equation (2.11) is a system of homogeneous linear equations for which the three variables *α_x_*, *α_y_* and *α_z_* have non-trivial solutions. Therefore, the determinant of the coefficients of the system of homogeneous linear equations (2.11) is equal to zero, and the stress state equation of the tetrahedral element *OABC* satisfies the formula:

2.12 $$\left|\begin{array}{ccc} \sigma_{x}-\sigma & \tau_{yx} & \tau_{zx}\\ \tau_{xy} & \sigma_{y}-\sigma & \tau_{zy}\\ \tau_{xz} & \tau_{yz} & \sigma_{z}-\sigma \end{array}\right|=0$$

Expanding the above equation gives:

2.13 $$\sigma^{3}-I_{1}\sigma^{2}+I_{2}\sigma -I_{3}=0$$

where,

2.14 $$\begin{array}{l} I_{1}=\sigma_{x}+\sigma_{y}+\sigma_{z}\\ I_{2}=\sigma_{x}\sigma_{y}+\sigma_{x}\sigma_{z}+\sigma_{y}\sigma_{z}-\tau_{xy}^{2}-\tau_{xz}^{2}-\tau_{yz}^{2}\\ I_{3}=\sigma_{x}\sigma_{y}\sigma_{\text{z}}-\sigma_{x}\tau_{yz}^{2}-\sigma_{y}\tau_{zx}^{2}-\sigma_{z}\tau_{xy}^{2}+2\tau_{yz}\tau_{zx}\tau_{xy} \end{array}\}$$

The principal stress, *σ_i_*, is calculated as follows:

2.15 $$\left.\begin{array}{c} \sigma_{1}=2\sqrt{-\frac{\;p}{3}}\cos\frac{w}{3}+\frac{1}{3}I_{1}\\ \sigma_{2}=2\sqrt{-\frac{\;p}{3}}\cos\frac{w+2\pi }{3}+\frac{1}{3}I_{1}\\ \sigma_{2}=2\sqrt{-\frac{\;p}{3}}\cos\frac{w+4\pi }{3}+\frac{1}{3}I_{1} \end{array}\right\}$$

wherein,

2.16 $$\left.\begin{array}{c} w=\arccos\left[-Q/2\sqrt{-{\left(\frac{\;p}{3}\right)}^{3}}\right]\\ \;p=-\frac{1}{3}I_{1}^{2}+I_{2}\\ Q=-\frac{2}{27}I_{1}^{3}+\frac{1}{3}I_{1}I_{2}-I_{3} \end{array}\right\}$$

#### 2.2.3. Direction of principal stress

The directional cosines of the principal stress vector, relative to the *Y*-axis and *Z*-axis, are:

2.17 $$\left.\begin{array}{c} m_{i}=\frac{B}{\sqrt{A^{2}+B^{2}+C^{2}}}\\ n_{i}=\frac{C}{\sqrt{A^{2}+B^{2}+C^{2}}} \end{array}\right\}$$

wherein,

2.18 $$\left.\begin{array}{c} A=\tau_{xy}\tau_{yz}-(\sigma_{y}-\sigma_{i})\tau_{zx}\\ B=\tau_{xy}\tau_{zx}-(\sigma_{x}-\sigma_{i})\tau_{yz},\\ C=(\sigma_{x}-\sigma_{i})(\sigma_{y}-\sigma_{i})-\tau_{xy}^{2} \end{array}i=1,\;2,\;3.\right\}$$

The inclination angle and azimuth angle of the principal stress, *σ_i_*, are calculated using equation (2.19).

2.19 $$\left.\begin{array}{c} \theta_{i}=\arcsin n_{i}\\ \beta_{i}=\arcsin\;(m_{i}/\sqrt{1-n_{i}^{2}}) \end{array}\right\}$$

where *θ_i_* is the inclination angle of the principal stress on face *OXY* (when greater than zero, it is called the elevation angle, and when less than zero it is called the depression angle); and *β_i_* is the angle between the projection of the principal stress in the *XOY* plane and the *X*-axis, with the clockwise direction being positive and the counterclockwise direction being negative, and the azimuth angle of the principal stress being calculated from the *X*-axis azimuth.

In summary, it can be concluded from equations (2.14)–(2.19) that, when using the AE method, it is necessary to determine at least six stress components for the measuring point during the *in situ* stress measurements, namely *σ_x_*, *σ_y_*, *σ_z_*, *τ_xy_*, *τ_zy_* and *τ_xz_*, where *τ_xy_*, *τ_zy_* and *τ_xz_* are obtained from equation (2.9).

## 3. Experimental method

### 3.1. Geologic background of the Baijiao coal mine

**Figure 5.**
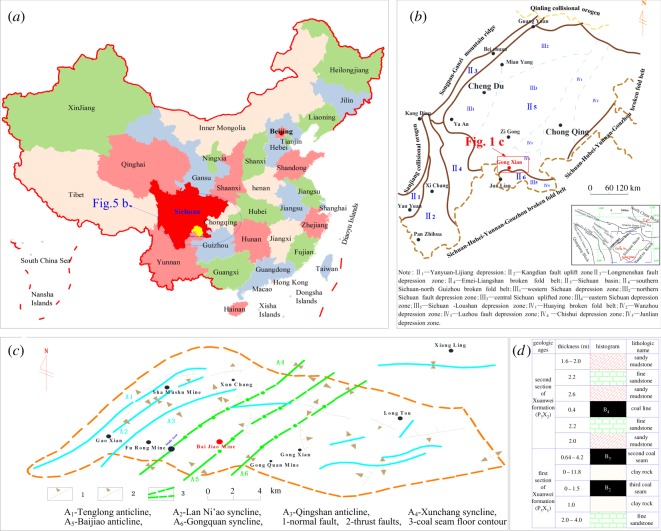
Location and geological structures. (*a*) Location of Baijiao coal mine. (*b*) Regional tectonic map of Baijiao Coal Mine. (*c*) The geological structural map of Baijiao Coal Mine. (*d*) Geological histogram of mining area.

The Baijiao coal mine is located in the southwest of Sichuan Province of China (figure 5*a*). It can be seen from the regional tectonic map (figure 5*b*) of mining area that the Baijiao Coal Mine is affected by multiple geological structures, such as the Yangtze plate, Qingling-Dabashan fault fold belt and Sichuan-Hubei-Yunnan-Guizhou fault fold belt, which cause the geological conditions of the coal seams to be extremely complex. It can be seen from the geological structural map (figure 5*c*) of the mine that the Baijiao mining area is mainly located on the west side of the Baijiao anticline, and is mainly affected by a series of north-northeast geological structures, such as the Tenglong anticline, the Lanniao syncline, and the Qingshan anticline, Xunchang syncline, and Gongquan syncline.

The coal-bearing stratum of the Baijiao mine is the Xuanwei formation, and there are three coal seams, B_2_, B_3_ and B_4_, which belong to the close-distance coal seam (figure 5*d*). Because the mining area experienced tectonic activity that affected the geologic structures, the tectonic coal developed widely, resulting in a high gas content and pressure, causing gas drainage difficulties and serious mine gas disaster potential [46]. The *in situ* stress as the basic parameters of coal and gas outburst risk analysis and roadway stability control research are of great significance to ensure the safe production of coal mine [47]. Therefore, the *in situ* stress measurement of the Baijiao mine is of great importance to analyse its coal and gas outburst disaster mechanisms.

### 3.2. Rock samples preparation

**Figure 6.**
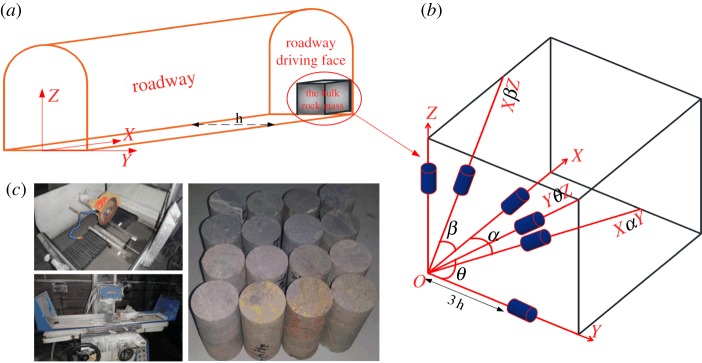
The rock specimen sampling and processing. (*a*) Schematic diagram of field sampling. (*b*) Schematic diagram of rock specimen sampling angle [48]. (*c*) Standard rock specimen processing.

The experimental rock samples were collected from the heading faces of the 2461 conveyor roadway of the B_2_ coal seam. The depth of the roadway is 476 m, the strike is NNW 330–340°, the dip direction is WSW 240–250° and the dip angle is 10–16°. Based on the construction conditions of the conveyor roadway's heading faces, the direction WSW 240° was defined as the *X*-axis; the direction NNW 330° was defined as the *Y*-axis; and the normal–normal direction of the XY surface (as defined herein) was defined as the *Z*-axis. The three-dimensional coordinate system is composed of the *X*, *Y* and *Z* axes (figure 6*a*). The rock samples were collected from six independent directions *X*-axis, *Y*-axis, *Z*-axis, X∠αY, X∠βZ and Y∠θZ (figure 6*b*). Wrapped in plastic wrap and sent to the laboratory for processing as standard rock specimens (φ50 × 100 mm) (figure 6*c*). In order to avoid secondary damage in the rock specimen processing process, in the rock samples collection process, the core drill pipe was used directly drilling specimens in tunnel surrounding rock. And the sampling depth is greater than three times the width of the roadway [49–51]. But because the surrounding rock is layered rock mass and has obvious bedding structure, some characteristics of bedding inevitably appear in the rock specimen.

### 3.3. Experimental equipment and experimental methods

**Figure 7.**
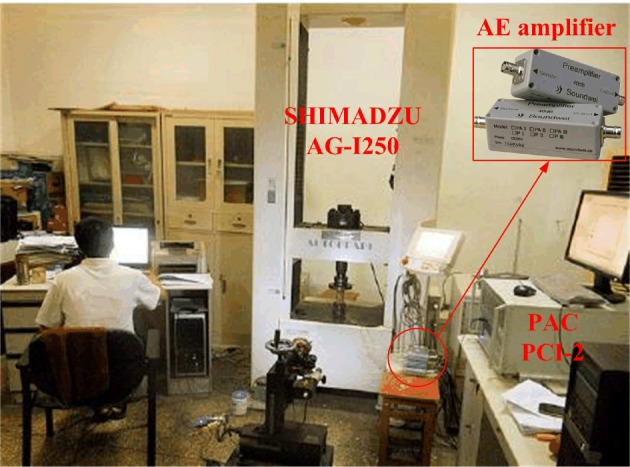
Experimental equipment.

**Figure 8.**
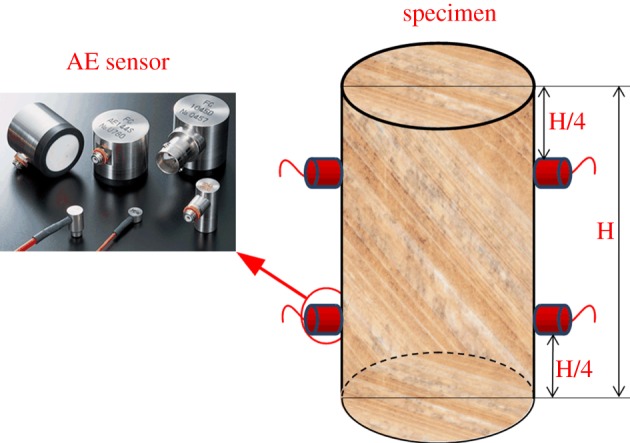
AE sensor installation diagram on the rock specimen.

The experimental equipment consisted of axial loading and AE monitoring systems. The axial loading system used a SHIMADZU AG-I250 electronic precision material testing machine, and the AE monitoring system used a PCI-2 AE system from the Physical Acoustic Corporation (figure 7). Before the experiment, Vaseline was used as a coupling agent to paste four AE transducers on the upper and lower ends of the specimen [52,53], as shown in figure 8. Then the sound emission signal channel function was checked, and the parameters for the AE collection and analysis system were set, the gain of the pre-amplifier was set to 6 dB, and the threshold of the AE transducer was set to 45 dB [52,54]. After confirming that the PCI-2 AE system was working properly, a uniform layer of grease was applied to the rock specimen's surface, the specimen was placed on the AG-I250 test bench, the AG-I250's loading head was adjusted so that it contacted the rock specimen's upper face, and the stress loading parameters were entered. The loading was controlled by displacement, and the loading speed was maintained at 0.02 mm min^−1^ until the rock was fractured or unstable, and then the loading was stopped. After the preparation work was completed, the AG-I250 axial loading system and the PCI-2 AE system were operated to initiate the experiment.

## 4. Results of experimental and *in situ* stress calculation

### 4.1 Mechanical properties of rock specimens

**Figure 9.**
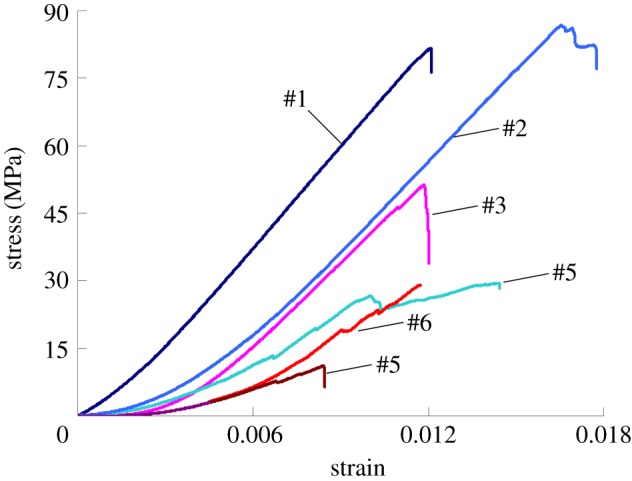
Stress–strain curves of rock specimens.

After the uniaxial compression testing (UCT) was completed, a representative set of the experimental results were selected for analysis. The stress–strain curves are shown in figure 9.

**Figure 10.**
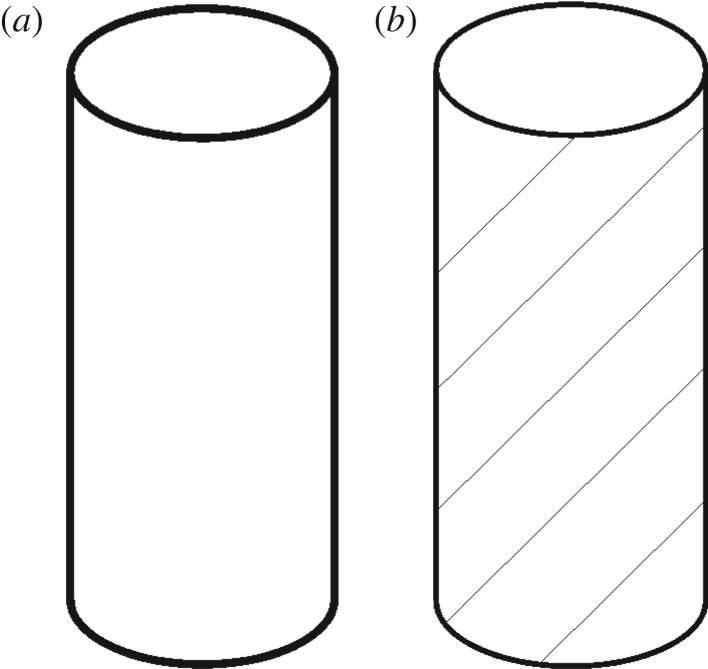
Schematic of two categories of rock specimens. (*a*) homogeneous rock samples (#1, #2, #3), (*b*) bedding rock samples (#4, #5, #6).

As we can see from the figure 9, the rock specimens from different directions have different failure modes and mechanical properties, and show obvious anisotropy. Because the rock specimens were drilled from a sedimentary rock series, the samples from the six directions have different bedding characteristics. According to the bedding characteristics of rocks, these rock specimens are divided into two categories, homogeneous rock samples (#1, #2, #3) and bedding rock samples (#4, #5, #6), As shown in figure 10. Because the rock's composition and structure significantly influence its mechanical properties, the non-homogeneous rock samples will inevitably have deviations in their mechanical properties, as seen for specimens #4, #5 and #6. Therefore, within the same rock mass, the bedding structure considerably influences the rock's internal damage from mechanical loads, resulting in large differences in the UCT and failure modes of the rock specimens in different directions, and explaining their anisotropic mechanical properties [55].

### 4.2. Acoustic emission characteristics of rock specimens

**Figure 11.**
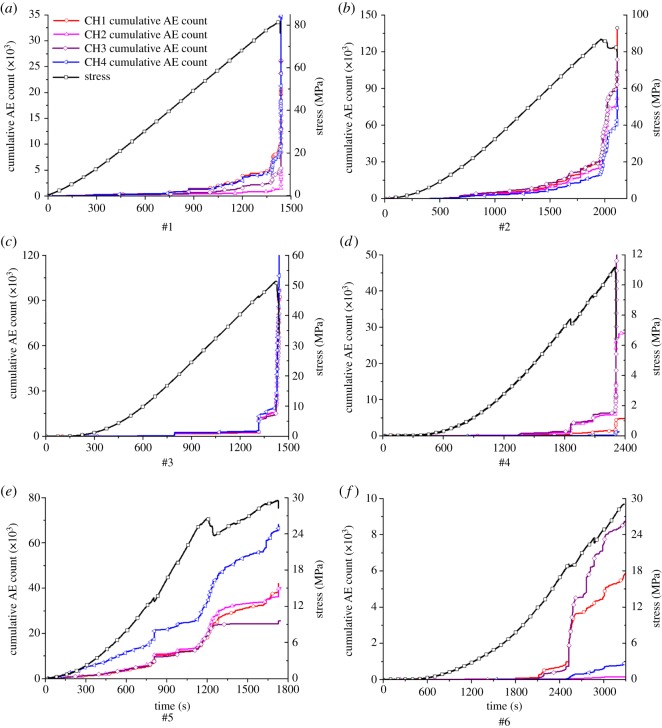
Relationship between the stress and the cumulative AE count, over time, of the rock specimens during the UCT process.

Through the analysis of the experimental AE and stress–strain data, curves representing the relationship between the stress and the cumulative AE count, over time, of the rock specimens during the UCT process were obtained, as shown in figure 11. The curves that show the relationships between the stress, cumulative AE count and time, as shown in figure 11*a*–*c*, are those from rock specimens #1, #2 and #3, respectively.

From these curves (figure 11*a*–*c*), the cumulative AE count received by the four AE transducers was approximately equal, showing that the homogeneous rock specimens demonstrated consistent change during the entire UCT process. During the pore fracture compaction stage, few AE counts were generated. As the stress was continuously increased, the AE count gradually increased, but the rate of the increase lessened until the axial load reached the peak strength when the cumulative AE count rose sharply. The entire process could be characterized as ‘slow increase → sharp increase’. The cumulative AE count of the #1 rock specimen showed a sharp increase phenomenon when the axial force was 99.4% of the peak strength, while the #2 and #3 specimens displayed this phenomenon at 99.5% and 99.9% of peak strength, respectively. Thus, the UCT failure process of the rock specimens was always followed by the generation of a transient AE count. The increasing trend of the cumulative AE count is positively correlated with the rock specimen's degree of internal damage. Before the failure and instability of the rock specimen, the AE count and its release rate reached their maximums.

The relationship curves of stress, cumulative AE count, and time for specimens #4, #5 and #6 (figure 11*d*–*f*) show that the AE counts received from the different channels of the bedding rock specimens were different, in both time and quantity, during the experiment, but the AE counts of the four channels, each corresponding to a specimen, had a nearly uniform trend, meaning that the AE during the failure process resulting from the uniaxial compression of the bedding rock differed spatially in quantity, but displayed the same change trend. It can be seen from figure 11*d*–*f* that the cumulative AE count for the bedding rock specimens showed an obvious ‘stepped’ rising pattern as the rock specimen developed internal damage. For example, sudden increases in the cumulative AE count of specimen #4 appeared at 35%, 69.1%, 82.4% and 99.6% of its peak compressive strength. The second and third rises occurred when the stress–time curve dipped, and the last occurred just prior to the rock's destruction. Sudden increases in the cumulative AE count of specimen #5 occurred at 45%, 90% and 98% of its peak strength, while for specimen #6, they occurred at 41.2%, 65.8% and 85.2% of its peak strength. Combined with the stress–time relationship curves, it can be seen that most of the sudden increases were related to the damage of the rock specimens.

In summary, the difference in the AE characteristics between homogeneous rock specimens and bedding rock specimens mainly manifested in: (i) the growth trend of the cumulative AE count with time had a ‘slow increase → sharp increase’ pattern for homogeneous rock, while the bedding rock showed a pattern of ‘stepped’ increase, (ii) regarding the differences between the AE counts during the failure process, the four channel transducers used for specimens #1, #2 and #3 were consistent with respect to the number and trend of the AE counts, while in the case of specimens #4, #5 and #6, there were spatial differences in the AE counts.

### 4.3 Kaiser point identified of rock specimens

**Figure 12.**
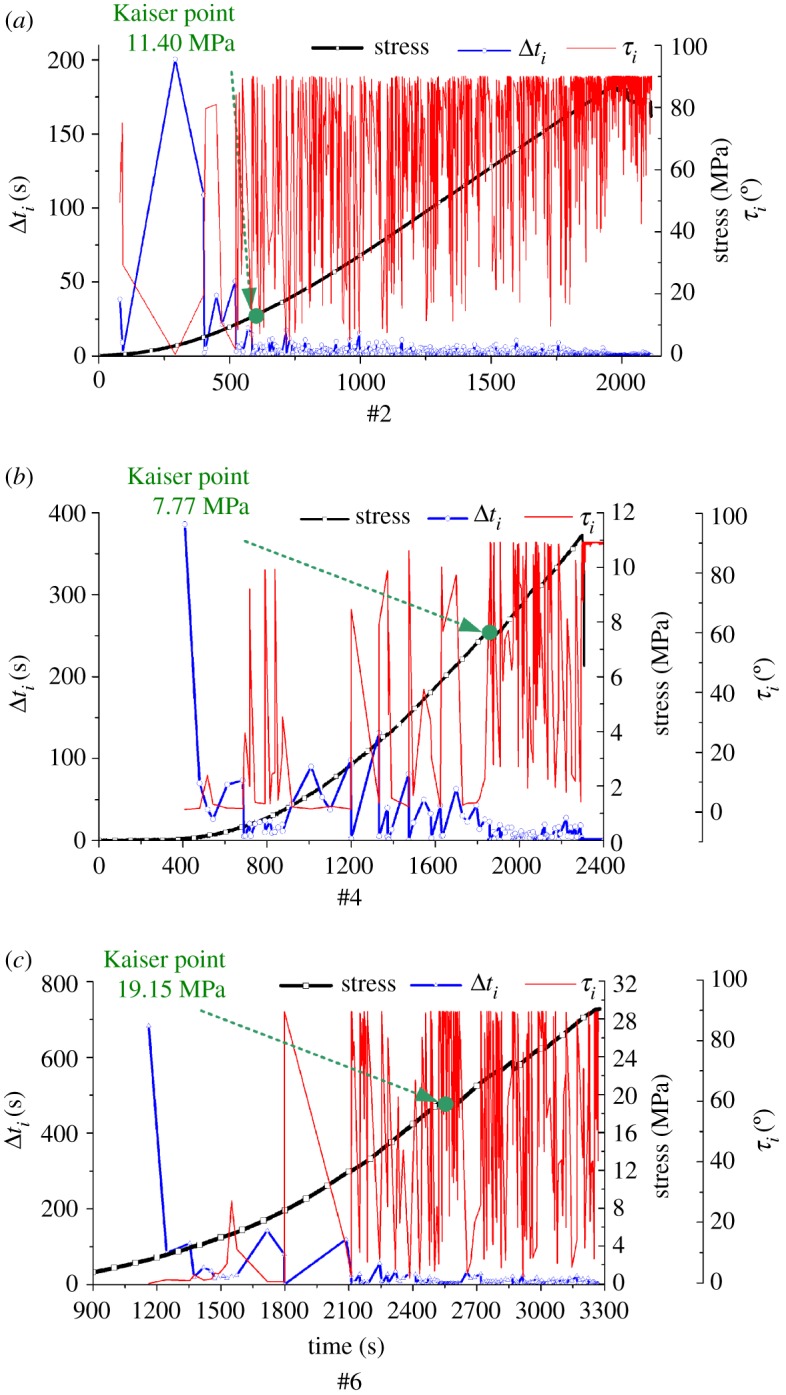
Curves representing the relationship between the stress, Δ*t_i_*, and *τ_i_* over time.

The Kaiser point of the rock samples in six directions, specimens #1–#6, were identified using the comprehensive method in §2.1. The result of the test data analysis of the rock specimens #2, #4 and #6 is shown in figure 12.

From the curve representing the relationship between the stress and cumulative AE count over time for rock sample #2, shown in figure 11*b*, the time of the Kaiser effect point was preliminarily determined to be before *t* = 700 s. Then, the curve representing the relationship between the stress, Δ*t_i_*, and *τ_i_* over time was obtained, shown in figure 12*a*, using the experimental AE parameters and equation (2.1). In order to analyse and determine the specific time of the Kaiser effect point and its corresponding stress value, the Δ*t_i_* and *τ_i_* parameters near the time of 700 are analysed according to figure 12*a*, and the analysis shows that when the time is 692.778 s, Δ*t_i_* = 0.002, *τ_i_* = 90.0. According to the analysis in §2.1, when the time is 692.778 s, the corresponding stress value (17.00 MPa) is the Kaiser effect point of the rock specimen in the *Y* direction. It can be seen from figure 11*d* that the approximate time of the Kaiser effect point for specimen #4 specimen was *t* = 1850 s. In addition, it can be seen from figure 12*b* that when the time is 1859.157 s, Δ*t_i_* = 0.04, *τ_i_* = 89.7. Therefore, the stress value (7.77 MPa) corresponding to this time is the Kaiser effect point of the rock specimen #4. For rock specimen #6, it can be seen from figure 11*f* that after the time is 2400 s, the cumulative calculation of the AE count produces a sharp increase. According to figure 12*c*, it can be seen that when the time is 2613.050 s, Δ*t_i_* = 0.011, *τ_i_* = 90.0. Therefore, the stress value (19.15 MPa) corresponding to this time is the Kaiser effect point of the rock specimen #6. Similarly, the Kaiser effect points of specimens #1, #3 and #5 were obtained using this method, and the maximum historical principal stresses of the rock specimen in six directions were obtained, as shown in table 1.

**Table 1.** Kaiser effect points of rocks specimens.

specimen number	specimen direction	time (s)	Δ*t_i_* (s)	*τ_i_* (**°**)	stress (MPa)
1#	*X*	281.490	0.113	90.0	11.50
2#	*Y*	692.778	0.002	90.0	17.00
3#	*Z*	796.264	0.020	90.0	19.10
4#	*X*∠45*Y*	1859.157	0.040	89.7	7.77
5#	*X*∠60*Z*	775.184	0.060	90.0	12.80
6#	*Y*∠45*Z*	2613.050	0.011	90.0	19.15

### 4.4 Results of principal stress using the novel method

Based on the information from table 1, the principal stress values and direction of working face 2461 of the Baijiao coal mine were calculated using equations (2.9), (2.12) and (2.14)–(2.19), as shown in table 2.

**Table 2.** Results of *in situ* stress determination using the Kaiser effect method.

contents	values (MPa)	inclination angle (**°**)	azimuth angle (**°**)
maximum principal stress	24.4	5.4	65.6
intermediate principal stress	17.1	−77.0	−0.2
minimum principal stress	6.1	11.8	−25.6

According to table 2, the maximum principal stress was 24.4 MPa; the inclination angle was 5.4°; the minimum principal stress was 6.1 MPa; and the inclination angle was 11.8°, all of which are close to the horizontal direction, represents the maximum main stress and minimum principal stress in this *in situ* stress measuring point are horizontal stress. And the ratio of maximum principal stress to minimum principal stress is 4 : 1, mainly induced by horizontal tectonic stress. The intermediate principal stress is 17.1 MPa, the inclination angle is 77°, close to the vertical direction. The maximum principal stress was 1.43 times greater than the middle principal stress, indicating that the *in situ* stress of working face 2461 in the Baijiao coal mine is mainly horizontal stress. The main direction of the principal stress is east-northeast (ENE); the direction of north-northwest (NNW) is secondary.

## 5. Discussion

### 5.1 Results verification by stress relief method

**Figure 13.**
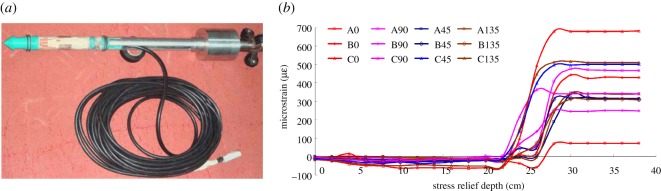
Instrument of stress relief method. (*a*) The hollow inclusion gauge; (*b*) deformation curve of stress relief process.

Field measurements were also performed using the stress relief method [10] to verify the results of the *in situ* stress values obtained using the AE method proposed in this study. The measurement instrument was a KX-81 hollow inclusion strain gauge (figure 13) and a KJ327-F mine pressure monitoring system developed by the Institute of Geomechanics of the Chinese Academy of Sciences. Microstrains were recorded during overcoring using the hollow inclusion strain gauge. The azimuth angle of the measurement hole was 20°, the dip angle was 4° and the depth was 10 m. After overcoring, biaxial tests [10] were performed to obtain the Young's modulus of 59 MPa, and the Poisson's ratio of 0.3. The results of the *in situ* stress measurements using the overcoring method are shown in table 3.

**Table 3.** Results of *in situ* stress determination using the overcoring method.

contents	values (MPa)	inclination angle (**°**)	azimuth angle (**°**)
maximum principal stress	24.3	−9.0	201.9
intermediate principal stress	17.3	−70.7	84.8
minimum principal stress	6.4	16.9	114.6

The results of *in situ* stress measured using the Kaiser effect and stress relief methods were compared, showing that the deviation rate of the maximum principal stress was: $(|\sigma_{1re}-\sigma_{1ae}|/\sigma_{1re})\times 100\text{\%}=0.4\text{\%}$; the deviation rate of the intermediate principal stress was: $(|\sigma_{1re}-\sigma_{1ae}|/\sigma_{1re})\times 100\text{\%}=1.2\text{\%}$; and the deviation rate of the minimum principal stress was: $(|\sigma_{1re}-\sigma_{1ae}|/\sigma_{1re})\times 100\text{\%}=4.9\text{\%}$. These results indicate that the two methods have little difference.

### 5.2 Results verification using hydraulic fracturing method

Stress measurements using the HF method were conducted on floor roadway 238. The equipment used was a CBYL400 fracturing pump group, jointly developed by the Chongqing Energy Investment Group Technology Co., Ltd and the Chongqing Pump Co., Ltd. This pump group has the advantages of large flow, small volume and high intelligence. The HF equipment was installed in the fresh airflow of the #3 cross drift. A high-pressure water pipe was laid between the #3 cross drift and floor roadway 238. The azimuth of the test hole was 185°, and the dip angle was 20°. Cement mortar was used for sealing, and 48 h after it completely solidified, the HF *in situ* stress test started.

**Figure 14.**
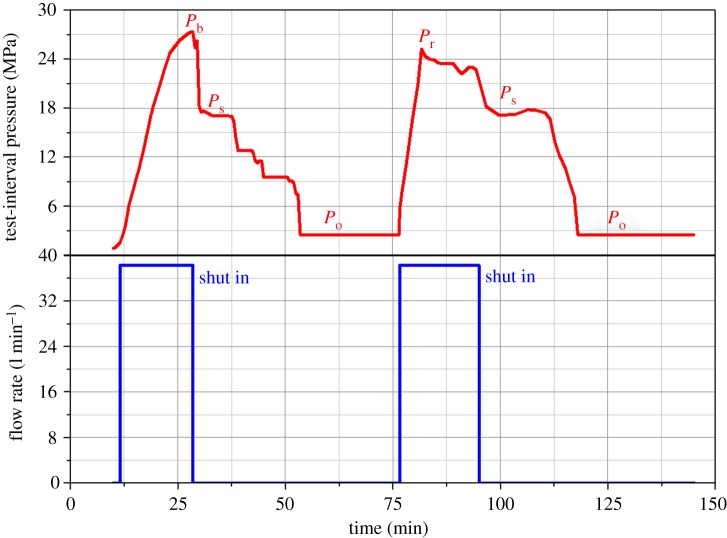
The curve of test-interval pressure and flow rate with time.

Figure 14 shows the curve representing the test-interval pressure and flow rate over time. The breakdown pressure *P*_b_ = 27.3 MPa; the fracture reopening pressure *P*_r_ = 25.2 MPa; the shut-in pressure *P*_s_ = 17.8 MPa; and the pore pressure *P*_0_ =2.48 MPa. Based on the above parameters, according to the study of Haimson & Fairhurst [17] and Zhao [56], the stress distribution of the borehole wall can be calculated. In this study, the stress distribution measured by the HF test was *σ_H_*_max_ = 25.72 MPa and *σ_H_*_min_ = 17.8 MPa.

Because the results calculated using the HF method represent the two-dimensional stress on the hole wall, they cannot be directly used to verify the accuracy of the three-dimensional stress results obtained using the AE method for *in situ* stress measurement. Therefore, based on the results of the three-dimensional principal stress calculation using the AE method and the azimuth angles of the boreholes used for the HF method, the stress distribution of the borehole wall was calculated through a series of coordinate transformations of the three-dimensional stress results giving results of $\sigma_{H\max}^{\prime }=24.4\;\mathrm{MPa}$  and $\sigma_{H\min}^{\prime }=17.1\;\mathrm{MPa}$. The deviation rate of the maximum horizontal stress *σ_H_*_max_ was: $(|\sigma_{H\max}-{\sigma^{\prime }}_{H\max}|/\sigma_{H\max})\times 100\text{\%}=5.1\text{\%}$, and the deviation rate of the minimum horizontal stress was: $(|\sigma_{H\min}-{\sigma^{\prime }}_{H\min}|/\sigma_{H\min})\times 100\text{\%}=3.9\text{\%}$.

## 6. Conclusion

*In situ* stress is one of the most important parameters for underground coal mining engineering. In response to the requirement of *in situ* stress measurement in the deep mining of coal mines in China, this paper put forward a calculation method for *in situ* stress measurement based on the Kaiser effect through theoretical research. The reliability of the *in situ* stress calculation method was verified by laboratory and field research. The results show that the method can be used for acoustic emissions *in situ* stress measurement.

(1) According to the *in situ* stress measurement method proposed in this study, the principal stress of Baijiao coal mine were calculated, and the *σ*_1_ = 24.4 MPa, *σ*_2_ = 17.1 MPa and *σ*_3_ = 6.1 MPa. And the ratio of maximum principal stress to minimum principal stress is 4 : 1, the maximum principal stress was 1.43 times greater than the middle principal stress.
(2) The *in situ* stress measurement results calculated by the novel method studied was verified by stress relief method and HF method; the measurement results are quite consistent. It indicates that the Kaiser effect identification comprehensive method developed in this paper can ensure the quality and reliability of test data.
(3) Under uniaxial compression testing, the failure processes of the rock specimens with no bedding were loading → complete failure, demonstrating brittle failure characteristics. While the failure processes of the rock specimens with bedding structures were loading → local shear → shear failure → loading → shear zone failure and their stress–strain curves showed multiple stress drop points. In terms of AE characteristics, the total AE counts of the rock specimens with no bedding increased with time, characterized as ‘slow growth → rapid growth’, while the bedding rock specimens showed a trend of ‘stepped’ increases.

## Data accessibility

We include all the experimental data in Dryad Digital Repository at: http://dx.doi.org/10.5061/dryad.b7r2510 [57].
